# ECMO-Therapien an einem peripheren Krankenhaus

**DOI:** 10.1007/s00063-025-01288-3

**Published:** 2025-05-23

**Authors:** Dominik J. Hoechter, Bernhard Oss, Martin Schmölz, Patrick Scheiermann

**Affiliations:** 1 Klinik für Anaesthesiologie, LMU-Klinikum, Ludwig-Maximilians-Universität München, Marchioninistr. 15, 81377 München, Deutschland; 2 Klinik für Anästhesie, Intensiv- und Notfallmedizin, Klinik Immenstadt, Klinikverbund Allgäu gGmbH, Immenstadt, Deutschland; 3 Fachbereich Anästhesie, Intensiv- und Schmerzmedizin, BG Unfallklinik Murnau, Murnau, Deutschland

**Keywords:** Extrakorporale Membranoxygenierung, ARDS, Fallzahlen, Krankenhausstrukturreform, Dezentrale Therapieangebote, Extracorporeal membrane oxygenation, ARDS, Caseload, Volume-outcome association, Decentralized healthcare

## Abstract

**Hintergrund:**

Extrakorporale Membranoxygenierung (ECMO) ist eine Therapieoption für anderweitig therapierefraktäre pulmonale bzw. kardiale Insuffizienz. Während die ECMO-Therapie als hochinvasives und risikoreiches Verfahren vornehmlich an spezialisierten Zentren vorgehalten wird, andererseits jedoch die Zeit zwischen Indikationsstellung und Initiierung der ECMO-Therapie relevant für das Behandlungsergebnis ist, stellt sich die Frage, ob auch in peripheren Krankenhäusern eine ECMO-Therapie sicher und erfolgreich eingesetzt werden kann.

**Methodik:**

Retrospektiv wurden an einem peripheren regionalversorgenden Krankenhaus über einen Zeitraum von 2013 bis 2023 alle Patienten erfasst, die eine ECMO-Therapie erhielten. Es wurden demografische Daten sowie Therapie- und Überlebensdaten erfasst.

**Ergebnisse:**

Im 10-jährigen Beobachtungszeitraum fanden an dem Zentrum 54 ECMO-Behandlungen statt (53 venovenöse ECMO, 1 venoarterielle ECMO), von denen 4 im weiteren Verlauf nach Therapieinitiierung an ein Zentrum verlegt wurden. Von den verbleibenden 50 Patienten überlebten 24 die intensivstationäre Therapie (48 %).

**Schlussfolgerung:**

Die vorliegende Untersuchung zeigt, dass auch an peripheren Krankenhäusern ECMO-Therapien sicher und mit gleichem Erfolg durchgeführt werden können, insbesondere wenn eine Anbindung an ein spezialisiertes Zentrum gewährleistet ist. So können Verlegungen an Zentren auf Patienten mit komplikativem Verlauf reduziert werden.

## Abstract (en)

**Introduction:**

Extracorporeal membrane oxygenation (ECMO) is a therapeutic option for otherwise refractory pulmonary or cardiac failure. While ECMO therapy, as a highly invasive and high-risk procedure, is primarily offered at specialized centers, the time between the indication for and the implementation of ECMO therapy is outcome-relevant. This raises the question of whether ECMO therapy can be safely and successfully implemented in peripheral hospitals.

**Methods:**

This retrospective analysis comprised all ECMO patients of a regional hospital for the period 2013–2023. Demographic data as well as therapy and survival data were recorded.

**Results:**

During the 10-year observation period, 54 ECMO treatments were performed at the center (53 venovenous ECMO, 1 venoarterial ECMO), of which four were transferred to a specialized center after the therapy was initiated. Of the remaining 50 patients, 24 survived the intensive care therapy (48%).

**Conclusion:**

The present study demonstrates that ECMO therapies can be performed safely and with similar outcomes at peripheral hospitals, particularly if supported by a collaborating specialized center. Thus, transfers to specialized centers can be limited to patients with complicated courses.

## Hintergrund

Technische Fortschritte und zunehmende Behandlungserfahrung bei Verfahren zur extrakorporalen Membranoxygenierung (ECMO) führten in den letzten Jahren weltweit zu einer zunehmenden Anwendung von ECMO: In Deutschland verzwölffachte sich die Fallzahl von venovenösen ECMO-Therapien (VV ECMO) schon vor der COVID-19-Pandemie im Zeitraum von 2007 bis 2018 [10].

Die beiden S3-Leitlinien „Invasive Beatmung und Einsatz extrakorporaler Verfahren bei akuter respiratorischer Insuffizienz“ und „Einsatz der extrakorporalen Zirkulation ECLS/ECMO bei Herz- und Kreislaufversagen“ empfehlen als Surrogatparameter für die Versorgungsqualität eine jährliche Mindestanzahl von 20 ECMO-Therapien [1, 5]. Diese wurde im Jahr 2018 deutschlandweit von nur 38 der insgesamt 231 Krankenhäuser (16 %), die ECMO-Therapien durchführten, erreicht [10].

Dem daraus abgeleiteten Bestreben nach Zentralisierung steht gegenüber, dass die Zeitdauer zwischen Indikationsstellung und Initiierung der ECMO-Therapie relevant für den Behandlungserfolg ist: Eine rasche Implementierung der Therapie nach Indikationsstellung ist mit höheren ECMO-Entwöhnungsraten und einer niedrigeren Mortalität assoziiert [14].

Vor dem Hintergrund der anstehenden Reformen im Gesundheitswesen mit weiter zunehmender Zentralisierung und Spezialisierung der Kliniken und bei stetig zunehmenden Übernahmeanfragen und limitierten Kapazitäten im Transport Schwerstkranker könnte die Initialisierung der ECMO-Therapie durch ein peripheres Krankenhaus mit sekundärer Zuverlegung in ein ECMO-Zentrum eine mögliche Strategie darstellen [18].

Diese retrospektive Studie untersucht, inwiefern und inwieweit ein niedrigschwelliger, zentrumsferner Zugang zur ECMO-Therapie möglich und sicher ist.

## Methodik

Nach Bescheinigung der ethischen Unbedenklichkeit durch die Ethikkommission der Medizinischen Fakultät der Ludwig-Maximilians-Universität, München, (Votum-Nr.: 24-0079) wurden über einen Zeitraum von 10 Jahren (2013–2023) mithilfe des Operations- und Prozedurenschlüssels alle Patienten identifiziert, die in einem regionalversorgenden Klinikverband (Klinikverbund Allgäu) eine ECMO-Therapie erhalten hatten. Es wurden demografische Parameter, klinische Daten sowie Behandlungs- und Überlebensdaten der Patienten erhoben und mit statistischen Standardmethoden explorativ dargestellt: Geschlecht, Alter, Diagnosen, Beatmungsdauer, Aufenthaltsdauer sowie Komplikationen. Methoden der deskriptiven Statistik wurden zur Beschreibung des Patientenkollektivs angewandt. Aufgrund der kleinen Fallzahl wurden die Patientendaten als nicht normalverteilt betrachtet. Sofern nicht anders angegeben wurde der Median und der Interquartilsabstand angegeben.

## Ergebnisse

Im Zeitraum von Januar 2013 bis einschließlich Mai 2023 wurden durch das ECMO-Team des Klinikum Immenstadt 54 Patienten (53 vv-ECMO, 1 va-ECMO) behandelt.

### Demografische Kennzahlen und ECMO-Indikationen

**Abb. 1:**
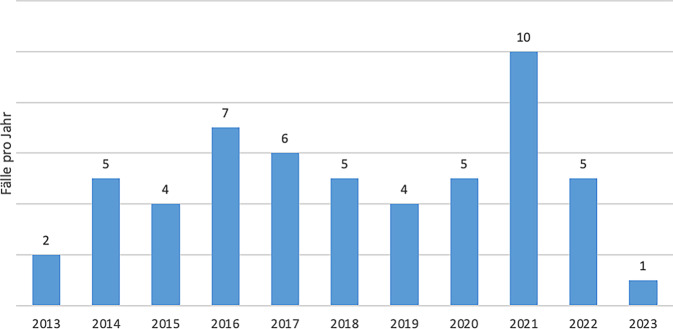
Jährliche ECMO-Fallzahlen im Klinikverbund Allgäu bis 01.06.2023

In der Mehrzahl der Jahre wurden zwischen 2 und 6 Therapien durchgeführt, im Jahr 2021 wurden 10 ECMO implementiert (Abb. 1).

Von den Patienten waren 14 weiblich (26 %) und 40 männlich (74 %). Die Patienten waren im Mittel 57 Jahre alt (47–64 a).

Die eine va-ECMO wurde bei einem Patienten mit beobachtetem, hypothermischem Kreislaufstillstand etabliert.

Bei den vv-ECMO-Therapien war mit 38 Fällen die häufigste Indikation eine Pneumonie, wobei virale Infekte – Influenza in 7 Fällen und SARS-CoV 2 in 15 Fällen – am häufigsten identifiziert werden konnten. Weitere Indikationen waren Aspirationspneumonien, infektexazerbierte chronische obstruktive Lungenerkrankung und Fälle von extrapulmonalem „acute respiratory distress syndrome“ (ARDS). Ursächlich bei extrapulmonalem ARDS waren Pankreatitiden, abdominelle Sepsis oder schwere Verletzungen und Lungenkontusion bei Polytrauma. In 4 Fällen wurde bei Pneumonitis bei Lungenfibrose die vv-ECMO-Therapie als überbrückende Therapie zur Lungentransplantation initiiert („*bridge to transplant*“).

In 30 Fällen (58 %) wurde die ECMO-Indikation bei schwerer Hypoxämie gestellt, in 4 Fällen (8 %) bei Hyperkapnie und 18 Patienten (35 %) befanden sich im respiratorischen Globalversagen.

Ein Patient mit fibrosierender Lungengerüsterkrankung wurde vor der vv-ECMO-Anlage nichtinvasiv druckunterstützt beatmet (NIV CPAP/ASB) und wurde wach an das ECMO-Gerät als Überbrückungstherapie zur Lungentransplantation („bridge to transplant“) angeschlossen; Patienten mit invasiver Beatmung wurden im Median 22 h (7,5–52 h) vor ECMO-Etablierung beatmet und der Oxygenierungsindex (Quotient aus arteriellem Sauerstoffpartialdruck [p_a_O_2_] und inspiratorischer Sauerstofffraktion [F_i_O_2_], sog. Horovitz-Quotient) vor vv-ECMO-Start lag im Median bei 72 mm Hg (58–88 mm Hg). Bei rein hypoxämischer respiratorischer Insuffizienz ohne begleitende pH-wirksame Hyperkapnie lag der Oxygenierungsindex vor ECMO-Anlage bei 64 mm Hg (52–80 mm Hg).

Der größte Anteil der Patienten (*n* = 26; 43 %) wurde vor ECMO-Therapie ausschließlich im Klinikum Immenstadt behandelt. Von diesen 26 Patienten wurden 20 notärztlich in der zentralen Notaufnahme vorgestellt – teils bereits mit der Diagnose der respiratorischen Insuffizienz bei Pneumonie oder Sepsis. 14 Patienten wurden intubiert und beatmet aus anderen Kliniken des Klinikverbunds nach Immenstadt zur weiteren Intensivtherapie verlegt.

13 Patienten wurden an unter ECMO aus anderen Kliniken im Klinikverbund nach Immenstadt transportiert. Dabei wurde je nach Verfügbarkeit mit einem Intensiv- oder Rettungshubschrauber (ITH/RTH), einem Intensivtransportwagen (ITW) oder auch mittels Rettungstransportwagen (RTW) verlegt.

### Behandlungserfolg und Komplikationen

Die mediane Dauer der ECMO-Therapien lag bei 10 Tagen (7–17 Tage). Die mediane Intensivverweildauer betrug 19 Tage (11–26 Tage). Vier der Patienten wurden im Verlauf unter ECMO in umliegende Zentren verlegt. Von den Verbleibenden 50 Patienten verstarben 26 (52 %) während der ECMO-Therapie: Ursächlich waren Komplikationen wie septisches Multiorganversagen, in jeweils einem Fall Exitus letalis bei hämorrhagischem Schock bei Hämatothorax und eine intrakranielle Blutung mit infauster Prognose: Zudem wurde bei einigen Patienten bei – zum Zeitpunkt der ECMO-Indikationsstellung unbekannten – prognosebestimmenden Nebendiagnosen (z. B. Erstdiagnose einer Neoplasie) eine Therapiezieländerung durchgeführt.

Bei insgesamt 20 Patienten kam es zu Komplikationen während der ECMO-Therapie (Tab. 1); 8 der Patienten mit einer ECMO-assoziierten Komplikation verstarben, wobei bei einem Patienten mit Hirnblutung unter ECMO-Therapie ein unmittelbarer Zusammenhang und bei 2 Patienten mit Lungenblutungen ein fraglicher Zusammenhang zwischen Komplikation und Todesursache (jeweils septisches Multiorganversagen) bestand.

**Tab. 1:** Übersicht über die Komplikationen. Bei 20 Patienten traten Komplikationen auf. Mehrfachnennungen vorhanden, z. B. Lungenarterienembolie bei HIT II

Komplikationen	
*Komplikationen bei der Kanülierung*	
Spannungspneumothorax	1
Perikarderguss	1
*Hämostaseologische Komplikationen*	
Gerinnungsaktivierung/DIC	3
HIT II	5
Heparinresistenz	1
Thrombose, venös	2
Lungenarterienembolie	1
Kolonischämie	1
Lungenblutung	2
Gastrointestinale Blutung	2
Schleimhautblutung/Blutungsneigung	2
Hämatothorax	1
Intrazerebrale Blutung	1
*Andere*	
Ileus	1
Herzrhythmusstörung (pVT)	1

*DIC* disseminierte intravasale Gerinnungsaktivierung (Koagulation), *HIT II* heparininduzierte Thrombopenie Typ II, *pVT* pulslose ventrikuläre Tachykardie

Bei einem Patienten trat im Rahmen der ECMO-Kanülenanlage einer Doppellumenkanüle ein Spannungspneumothorax und ein Perikarderguss auf. Der Großteil der Komplikationen betraf Störungen der Blutgerinnung: Bei 3 Patienten wurde eine disseminierte intravaskuläre Gerinnungsaktivierung (DIC), bei 4 Patienten eine heparininduzierte Thrombopenie (HIT) Typ II diagnostiziert. Bei 4 Patienten ereigneten sich thromboembolische Ereignisse: bei 2 Patienten zeigte sich eine Teilthrombosierung der Vena jugularis interna, in der jeweils eine Kanüle platziert war, ein Patient erlitt eine Lungenarterienembolie und ein Patient eine Darmischämie. Die Lungenarterienembolie und eine venöse Thrombose traten bei Patienten mit der Diagnose einer HIT II auf. Ein Patient zeigte Zeichen einer Heparinresistenz (Notwendigkeit außergewöhnlich hoher Heparindosierungen zur Erreichung der Ziel-aPTT, ggf. mit vermehrter Gerinnselbildung im extrakorporalen Kreislauf).

Zwei Patienten wiesen eine diffuse Blutungsneigung mit spontanen Schleimhautblutungen und Blutungen aus den Kanüleneinstichstellen auf. Jeweils 2 Patienten erlitten gastrointestinale bzw. pulmonale Blutungen. Ein Patient entwickelte einen Hämatothorax. Ein Patient musste bei Ileus laparotomiert und darmreseziert werden; ein Patient zeigte eine Episode einer pulslosen Kammertachykardie unklarer Ätiologie und konnte reanimiert werden.

16 Patienten (32 %) konnten nach erfolgreichem ECMO-Weaning auf eine Normalstation verlegt werden, 8 Patienten (16 %) wurden direkt von der Intensivstation in eine neurologische Frührehabilitations- oder Weaning-Klinik verlegt.

Neben diesen 54 Patienten wurden im Beobachtungszeitraum weitere 4 Patienten von mobilen ECMO-Teams umliegender Zentren kanüliert und unmittelbar nach Anlage von diesen unter laufender ECMO-Therapie verlegt. Drei dieser Patienten wurden mit einer va-ECMO bei kardiogenem bzw. gemischt septisch-kardiogenem Schock bei septischer Kardiomyopathie bei Waterhouse-Friedrichsen-Syndrom versorgt, ein Patient wurde bei akutem Lungenversagen (ARDS) mit vv-ECMO versorgt.

## Diskussion

Die vorliegende Untersuchung analysiert deskriptiv die Durchführung von ECMO-Verfahren an einem peripheren Krankenhaus am Beispiel eines Regionalversorgers im Allgäu. Diese ländlich-alpin geprägte Region liegt im bayerischen Südwesten. Da im Bereich des Allgäus kein Intensivtransportmittel stationiert ist, muss bei Verlegungen kritisch kranker Patienten neben der Entfernung von 120–150 km zu den nächsten (universitären) Krankenhäusern überregionaler Versorgungsstufe immer auch die Anfahrts- oder Anflugszeit berücksichtigt werden. Zudem können auch die alpine Topografie gemeinsam mit klimatischen Faktoren, wie Nebel, niedriger Wolkendecke oder Schneefall, den raschen und schonenden Patiententransport zu Land und zu Luft limitieren, selbst wenn diese Transportmittel ubiquitär vorhanden wären [7, 13].

Die vorliegende Datenanalyse demonstriert, dass auch an einem peripheren Zentrum ECMO-Therapien sicher durchgeführt werden können, wenn eine rechtzeitige Verlegung der Patienten nicht möglich ist. Im Beobachtungszeitraum wurden in nur 4 Fällen Patienten von ECMO-Teams anderer Zentrumskliniken kanüliert und unter laufender ECMO-Therapie direkt in die jeweiligen Krankenhäuser transportiert. Von den 54 Patienten, die vom ECMO-Team vor Ort versorgt wurden, wurden lediglich 4 im weiteren Verlauf der ECMO-Therapie sekundär in andere Kliniken verlegt.

Nationale und internationale Leitlinien empfehlen die Indikation einer vv-ECMO-Therapie bei einem Oxygenierungsindex < 80 mm Hg oder einer relevanten Hyperkapnie (pH < 7,25 mit arteriellen Kohlendioxidpartialdruckwerten [p_a_CO_2_] > 60 mm Hg; [1, 21]). In der Patientenkohorte lag der Oxygenierungsindex nach Horovitz vor vv-ECMO-Anlage im Median bei Werten zwischen 64 und 72 mm Hg und alle 4 Patienten mit hyperkapnischem Lungenversagen zeigten pH-Werte < 7,25.

Zudem wurde bei Indikationsstellung darauf geachtet, dass ECMO als Rescue-Verfahren nach Ausschöpfung anderer Behandlungsmaßnahmen, wie „Best-PEEP-Strategie“ und Bauchlagerung sowie inhalativer Applikation von Inodilatoren, eingesetzt wird.

Die bei dieser leitlinienkonformen Indikationsstellung resultierende Patientengruppe mit einem medianen Alter der Patienten von 57 Jahren wie auch einem Anteil von knapp einem Drittel weiblicher Patienten ist im Einklang mit den Beobachtungen einer nationalen Erhebung von ECMO-Therapien durch Friedrichson und Kollegen, die zeigten, dass die meisten ECMO-Patienten zwischen 55 und 65 Jahre alt waren und der Anteil weiblicher Patienten bei etwas über 30 % lag [10]. Auch andere große Querschnittsstudien im Bereich der vv-ECMO-Therapie, wie z. B. der EOLIA-Trial, beschrieben ähnliche Patientenkollektive mit einem mittleren Alter von 53 Jahren und etwas weniger als ein Drittel weiblicher Patienten [6].

Darüber hinaus war auch der Therapieerfolg mit einem Krankenhausüberleben von 53 % mit dem anderer publizierter Kohorten vergleichbar: Während die Zahlen des internationalen Registers der internationalen Fachgesellschaft für extrakorporale Zirkulationsverfahren (*Extracorporeal Life Support Organization*, ELSO) eine Gesamtmortalität von 39–47 % bei vv-ECMO angeben, wurde die Letalität von vv-ECMO-Patienten in Deutschland bei 55,6 % angegeben [8, 10, 20]. In der Subgruppe der ECMO-Therapien bei Lungenversagen aufgrund von SARS-CoV-2-Infektion lag die Sterblichkeit in der Kohorte von Immenstadt mit 44 % wesentlich unterhalb der gesamtdeutschen Letalität von 68 % [9, 12].

Gleichsam entsprechen die in der Kohorte berichteten Komplikationen in Art und Inzidenz dem Durchschnitt bei ECMO-Therapien:

Im Jahr 2013 veröffentlichten Zangrillo und Kollegen eine Metaanalyse von Komplikationen bei ECMO-Therapien, die 12 Studien mit über 1700 Patienten einschloss [22]. Blutungskomplikationen waren hier in etwas über 30 %, gastrointestinale Blutungen in 7 % der Fälle berichtet worden. Die Inzidenz venöser Thrombosen wurde mit 10 % ermittelt; eine intravasale Gerinnungsaktivierung wurde bei 5 % der Patienten festgestellt.

Heparinresistenz ist ein bei ECMO-Patienten, die in der Regel eine therapeutische Antikoagulation benötigen, häufig auftretendes Phänomen [17, 19]. Bei in der Literatur uneinheitliche verwendeten Definitionen und Grenzwerten schwanken die Inzidenzangaben zwischen 0 und 60 %. In einer aktuellen Arbeit von Nagler und Kollegen wurde diese mit 17 % angegeben. Zudem wurde ein erhöhter Anteil von COVID-Patienten unter den Patienten mit Heparinresistenz festgestellt [17].

### Strukturanforderungen bei ECMO-Therapie

Um bestmögliche Behandlungsergebnisse im Bereich der bis heute komplikationsträchtigen ECMO-Therapien zu erzielen, werden neben patientenindividuellen Faktoren immer wieder auch strukturelle Anforderungen an die Intensivtherapieeinheiten gestellt. In der S3-Leitlinie „Invasive Beatmung und Einsatz extrakorporaler Verfahren bei akuter respiratorischer Insuffizienz“ werden hier unter anderem eine leistungsfähige Blutbank, ein Zentrallabor, die Möglichkeit der computertomographischen Untersuchung und notfallmäßige sofortige Verfügbarkeit von Gefäß‑, Abdominal- und Thoraxchirurgie sowie die regelmäßige Anwendung von ECMO gefordert [1]. Bei letzterem verzichtet die Leitlinie auf die ausdrückliche Nennung einer Mindestfallzahl, schließt sich jedoch anderen internationalen Empfehlungen von mindestens 20 ECMO-Therapien pro Jahr, davon 12 bei Lungenversagen, an [1].

Allerdings konnten Barbaro und Kollegen in einer Auswertung des internationalen ELSO-Registers (7759 Patienten im Zeitraum von 2008–2013) keinen statistisch signifikanten Zusammenhang von Jahresfallzahl und Patientenüberleben bei vv-ECMO oder ARDS-Patienten zeigen [2].

McCarthy und Kollegen untersuchten ebendiese Fragestellung auf Grundlage des National (Nationwide) Impact Sample (NIS) der USA über den Zeitraum von 2002–2011. Sie zeigten und publizierten Mortalitätsraten von 48 %, 60 % bzw. 57 % für Zentren niedriger, mittlerer bzw. hoher Fallzahlen [16]. Übereinstimmend zeigte eine retrospektive Analyse zweier Zentren in Queensland, Australien, die gemeinsam 49 vv-ECMO-Therapien in einem 10-Jahres-Zeitraum durchgeführt haben, sehr gute Überlebensraten (84 %) der dort behandelten Patienten [11].

Die aus anderen Bereichen der Medizin bekannte Assoziation von höheren Fallzahlen mit besserem Outcome scheint sich also nicht ohne weiteres auf den Bereich der vv-ECMO-Therapie übertragen zu lassen.

Die Evidenz für die immer wieder – sei es in Publikationen, Zertifikaten der Fachgesellschaften oder der S3-Leitlinie – in den Raum gestellte Mindestfallzahl von 20 ECMO-Therapien pro Jahr scheint schwach; eine solche Richtzahl birgt darüber hinaus die Gefahr der potenziell zu frühen oder zu großzügigen Indikationsstellung bei marginaler Indikation für das Verfahren [1, 3, 4].

### Bedeutung von Ausbildung und Erfahrung des Behandlungsteams

Von Bedeutung ist zweifelsohne die Ausbildung und Erfahrung der Behandlungsteams, denen es gelingen kann, auch an peripheren Krankenhäusern solche Therapieprogramme in einer Art *Bottom-to-top*-Ansatz zu etablieren. Gleichsam wurde das ECMO-Programm am Klinikum Immenstadt zum einen aus der Notwendigkeit heraus aufgebaut, dass nicht immer eine Versorgung mit ECMO aus einem Zentrum für die Patienten verfügbar war. Zum anderen arbeiteten auf der Intensivstation des Klinikums Immenstadt – sowohl im ärztlichen wie auch pflegerischen Dienst – mehrere Kollegen mit langjähriger Erfahrung im Bereich der ARDS- und ECMO-Therapie. Im ECMO-Team des Krankenhauses sind 5 Fachärzte der Anästhesiologie bzw. Inneren Medizin, die alle die Zusatzbezeichnung *spezielle Intensivmedizin* innehaben und während ihrer Ausbildung an Kliniken der Maximalversorgung bereits Erfahrung in der Versorgung von ECMO-Patienten sammeln konnten. Zudem sind ausgewählte Pflegekräfte mit Fachweiterbildung in Anästhesie und Intensivmedizin im Aufbau und Priming der Konsole geschult. Alle Ärzte und Pflegekräfte, die auf der Intensivstation arbeiten, sind auf die ECMO-Konsole eingewiesen und erhalten jährlich ein 1,5-tägiges interaktives Training zu Grundlagen der ECMO-Therapie und Problemlösung an der Konsole.

Das Krankenhaus verfügt neben einer Abteilung für Allgemein- und Viszeralchirurgie auch über eine gefäßchirurgische Abteilung mit einem modernen Hybridoperationssaal für endovaskuläre Eingriffe sowie eine interventionelle Kardiologie. So werden die wesentlichen, in den Leitlinien genannten Strukturvoraussetzungen erfüllt.

Eine Vergabe von Versorgungsaufträgen im Rahmen der Krankenhausstrukturplanung – basierend auf einer Auswertung der Behandlungsergebnisse und bestehender Strukturen – könnte dazu beitragen, dass auch kleinere Kliniken im ländlichen Raum berücksichtigt werden.

Dies könnte Kliniken mit nachweislich hoher Behandlungsqualität und geeigneten Strukturen stärken und gleichzeitig eine bedarfsgerechte sowie qualitätsgesicherte Patientenversorgung fördern.

Im Fall besonderer Fragestellungen oder komplikativer Verläufe könnten solche Programme zukünftig durchaus auch von (tele-)intensivmedizinischen Kooperationen mit spezialisierten Zentren profitieren [15]. Dies würde einen wesentlichen Beitrag dazu leisten, der Bevölkerung in zentrumsfernen Gebieten einen zeitnahen Zugang zu solchen spezifischen Therapien wie der ECMO zu gewährleisten.

## Zusammenfassung

Diese Erhebung zeigt, dass auch an einem peripheren Krankenhaus mit niedriger Fallzahl ECMO-Behandlungen sicher und mit gleichem Therapieerfolg möglich sind. Von der zeitnahen Implementierung des Verfahrens vor Ort können Patienten profitieren. Ressourcenaufwendige, ggf. zeitkritische Patiententransporte und die Kapazitäten spezialisierter Zentren können auf indizierte Fälle fokussiert werden.

## Fazit für die Praxis

- An einem peripheren Krankenhaus konnte ECMO-Therapie in einem Bottom-to-top-Ansatz sicher durchgeführt werden – auch wenn die empfohlenen Mindestbehandlungszahlen nicht erreicht werden.
- Behandlungserfolg und Komplikationsraten waren mit dem nationalen Durchschnitt vergleichbar.
- Patienten können von der zeitnahen Implementierung der ECMO-Therapie profitieren. Aufwendige Transporte können auf indizierte Fälle reduziert werden.

## Data Availability

Die erhobenen Datensätze können auf begründete Anfrage in anonymisierter Form beim korrespondierenden Autor angefordert werden. Die Daten befinden sich auf einem Datenspeicher an der Klinik für Anaesthesiologie.

## References

[1] Adamzik M, Bauer A, Bein T et al (2017) S3-Leitlinie Invasive Beatmung und Einsatz extrakorporaler Verfahren bei akuter respiratorischer Insuffizienz. https://register.awmf.org/assets/guidelines/001-021l_S3_Invasive_Beatmung_2017-12.pdf. Zugegriffen: 3. Okt. 2024

[2] Barbaro RP, Odetola FO, Kidwell KM et al (2015) Association of hospital-level volume of extracorporeal membrane oxygenation cases and mortality. Analysis of the extracorporeal life support organization registry. Am J Respir Crit Care Med 191:894–901 25695688 10.1164/rccm.201409-1634OC PMC4435456

[3] Bein T, Karagiannidis C, Weber-Carstens S et al (2022) ECMO-Einsatz bei COVID-19: Hohe Sterblichkeit in der Klinik. https://www.aerzteblatt.de/archiv/222958/ECMO-Einsatz-bei-COVID-19-Hohe-Sterblichkeit-in-der-Klinik. Zugegriffen: 15. Aug. 2024

[4] Bickenbach J, Moerer O, Dembinski R et al (2024) Modulares Zertifikat Intensivmedizin der DGAI. Anästhesiol Intensivmed. 10.19224/ai2024.381

[5] Boeken U, Ensminger S, Assmann A et al (2021) S3-Leitlinie Einsatz der extrakorporalen Zirkulation (ECLS/ ECMO) bei Herz- und Kreislaufversagen. Med Klin Intensivmed Notfmed 116:678–686 34665281 10.1007/s00063-021-00868-3

[6] Combes A, Hajage D, Capellier G et al (2018) Extracorporeal membrane oxygenation for severe acute respiratory distress syndrome. N Engl J Med 378:1965–1975 29791822 10.1056/NEJMoa1800385

[7] Dahmen J, Bouillon B, Royko M, Karagiannidis C (2023) Flächendeckende Notfallreform: Luftrettung maßgeblicher Faktor. Dtsch Ärztebl

[8] Extracorporeal Life Support Organization (2024) ELSO International Summary of Statistics. https://www.elso.org/registry/internationalsummaryandreports/internationalsummary.aspx. Zugegriffen: 15. Aug. 2024

[9] Friedrichson B, Kloka JA, Neef V et al (2022) Extracorporeal membrane oxygenation in coronavirus disease 2019: A nationwide cohort analysis of 4279 runs from Germany. Eur J Anaesthesiol 39:445–451 35180152 10.1097/EJA.0000000000001670

[10] Friedrichson B, Mutlak H, Zacharowski K, Piekarski F (2021) Insight into ECMO, mortality and ARDS: a nationwide analysis of 45,647 ECMO runs. Crit Care 25:38 33509228 10.1186/s13054-021-03463-2 PMC7841040

[11] Joyce CJ, Cook DA, Walsham J et al (2020) Low volume ECMO results study. Crit Care Resusc 22:327–334 38046879 10.51893/2020.4.OA5 PMC10692512

[12] Karagiannidis C, Slutsky AS, Bein T et al (2021) Complete countrywide mortality in COVID patients receiving ECMO in Germany throughout the first three waves of the pandemic. Crit Care 25:413 34844657 10.1186/s13054-021-03831-y PMC8628273

[13] Klier M, Prim AIRKonsortium, Wanka-Pail ER et al (2015) Luftrettung rund um die Uhr – Welchen Einfluss hat das Wetter?: Eine Untersuchung im Rahmen des Forschungsprojekts PrimAIR. Notf Rett Med 18:130–138

[14] Li X, Hu M, Zheng R et al (2021) Delayed initiation of ECMO is associated with poor outcomes in patients with severe COVID-19: A multicenter retrospective cohort study. Front Med 8:716086 10.3389/fmed.2021.716086 PMC8481658 34604257

[15] Marx G, Markewitz A, van Aalst G (2021) Telemedizin in der Intensivmedizin S1 Leitlinie der DGAI (001-034). https://register.awmf.org/assets/guidelines/001-034l_S1_Telemedizin_in-der-Intensivmedizin_2021-01_1.pdf. Zugegriffen: 3. Okt. 2024

[16] McCarthy FH, McDermott KM, Spragan D et al (2016) Unconventional volume-outcome associations in adult extracorporeal membrane oxygenation in the United States. Ann Thorac Surg 102:489–495 27130248 10.1016/j.athoracsur.2016.02.009 PMC5835397

[17] Nagler B, Staudinger T, Schellongowski P et al (2024) Incidence of heparin resistance and heparin failure in patients receiving extracorporeal membrane oxygenation: an exploratory retrospective analysis. J Thromb Haemost 22:2773–2783 38925491 10.1016/j.jtha.2024.06.008

[18] Puślecki M, Ligowski M, Dąbrowski M et al (2019) Development of regional extracorporeal life support system: The importance of innovative simulation training. Am J Emerg Med 37:19–26 29699897 10.1016/j.ajem.2018.04.030

[19] Raghunathan V, Liu P, Kohs TCL et al (2021) Heparin resistance is common in patients undergoing extracorporeal membrane oxygenation but is not associated with worse clinical outcomes. Asaio J Publ Ahead Print: 899–906 10.1097/MAT.0000000000001334 PMC9019066 33528163

[20] Rali AS, Abbasi A, Alexander PMA et al (2024) Adult highlights from the Extracorporeal Life Support Organization registry: 2017–2022. ASAIO J 70:1–7 37755405 10.1097/MAT.0000000000002038

[21] Tonna JE, Abrams D, Brodie D et al (2021) Management of adult patients supported with venovenous extracorporeal membrane oxygenation (VV ECMO): Guideline from the extracorporeal life support organization (ELSO). ASAIO J 67:601–610 33965970 10.1097/MAT.0000000000001432 PMC8315725

[22] Zangrillo A, Landoni G, Biondi-Zoccai G et al (2013) A meta-analysis of complications and mortality of extracorporeal membrane oxygenation. Crit Care Resusc 15:172–178 23944202

